# Au–Pd core–shell nanoparticles for enhanced catalytic performance in liquid-phase selective hydrogenation

**DOI:** 10.1039/d5cy00889a

**Published:** 2025-10-14

**Authors:** Marta Perxés Perich, Kristiaan H. Helfferich, Petra E. de Jongh, Jessi E. S. van der Hoeven

**Affiliations:** a Materials Chemistry and Catalysis, Debye Institute for Nanomaterials Science, Utrecht University 3584 CG Utrecht The Netherlands j.e.s.vanderhoeven@uu.nl

## Abstract

Au–Pd core–shell nanoparticles are promising selective hydrogenation catalysts and can exhibit strongly enhanced catalytic activities compared to their alloyed and monometallic counterparts, while retaining high selectivity. However, little is known about their performance and structural stability during liquid-phase selective hydrogenation. Here, we test colloidally synthesized Au–Pd core–shell and alloyed nanoparticles for the selective hydrogenation of 2-methyl-3-butyn-2-ol to 2-methyl-3-buten-2-ol, a model reaction for selective hydrogenation of alkynols to alkenols used in vitamin and fragrance synthesis. The core–shell nanoparticles were significantly more active than their alloy counterparts and also more selective. Moreover, they also outperformed their Au and Pd monometallic counterparts, with the core–shell nanoparticles being ∼3.5× more active than monometallic Pd while retaining its selectivity. This work shows how the use of structure-controlled colloidal core–shell nanoparticles can be useful to enhance the performance in liquid-phase selective hydrogenation catalysis.

## Abstract 2

Au–Pd core–shell nanoparticles outperform monometallic and alloyed counterparts in the liquid-phase selective hydrogenation of MBY.

## Graphical abstract

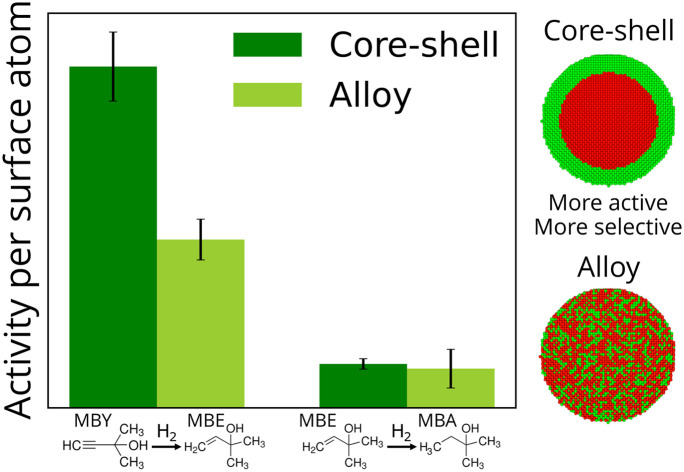

## Introduction

Selective hydrogenation of alkynes is key for many industrial processes, from the purification of alkene feeds after steam cracking^1,2^ to the production of fine chemicals.^3^ The challenge is to selectively convert alkynes to alkenes while preventing further hydrogenation to alkanes.^4^ The hydrogenation of the C–C triple bond in alkynols to a C–C double bond in alkenols is a step in the production process of fine chemicals,^3,5,6^ and is usually performed in the liquid-phase in a H_2_ pressurized batch-reactor.^7^ A prime example in this class of reactions is the selective hydrogenation of MBY (2-methyl-3-butyn-2-ol) to MBE (2-methyl-3-buten-2-ol), used for the production of vitamins and fragrances,^8^ while preventing the undesired full hydrogenation to MBA (2-methylbutan-2-ol).

Selective alkyne hydrogenation reactions are typically catalyzed by supported palladium (Pd) nanoparticles, as Pd enables rapid hydrogen dissociation at low temperatures.^4^ However, maintaining high selectivity towards the semi-hydrogenated alkene products is challenging, especially at low alkyne concentrations.^1,4,9^ A typical approach for enhancing alkene selectivity involves poisoning non-selective catalyst sites, a strategy used in Lindlar catalysts, where additives like lead and quinoline are introduced.^10,11^ Apart from the negative environmental impact of such toxic compounds,^3^ this poisoning strategy results in less effective Pd utilization,^4^ drastically decreasing the catalytic activity, and sometimes also lowering catalyst stability.^3^ Another strategy to improve the selectivity of the catalysts is to combine Pd with more selective coinage metals like Au, Ag or Cu.^4,12,13^ In these bimetallic nanoparticles, dilute Pd atoms and ensembles provide the active sites for hydrogen dissociation and hydrocarbon binding, while the vicinity of Au, Ag or Cu increases the selectivity by modifying the electronic properties of the Pd ensembles.^4^ This electronic effect is stronger in Au compared to Ag or Cu.^4^ The exact Pd ensemble size, which is set *via* the Pd/Au ratio, has a strong effect on the overall performance of the bimetallic nanoparticles.^14^ Dilute Pd alloys in Au (<10 atm% Pd) typically show the best compromise between activity and alkene selectivity.^15–17^ However, regardless of the composition, the performance of Au–Pd alloys follows an inherent activity-selectivity trade-off, where lowering of the Pd fraction increases the selectivity but unavoidably lowers the activity.

With the recent advances in material synthesis, it is possible to prepare complex bimetallic nanoparticles and control the location of the Au and Pd atoms. A prime example are core–shell nanoparticles, where one metal resides in the core and the other metal forms a shell around it. Interestingly, Au-core Pd-shell nanoparticles can overcome the activity-selectivity trade-off. Recently, our group has demonstrated that Au–Pd core–shell nanorods show up to 50 times higher activity than their alloy counterparts in the gas-phase selective hydrogenation of butadiene, while retaining high selectivity.^18^ Similarly, Au–Pd core–shell nanoparticles have shown enhanced activity compared to the monometallic and alloyed counterparts in selective oxidation catalysis, such as the selective oxidation of benzyl alcohol.^19–21^ Although the potential of Au-core and Pd-shell catalysts has been demonstrated in selective oxidation catalysis and gas-phase hydrogenation catalysis, they have remained unexplored in liquid-phase hydrogenation. The milder reaction temperatures used in liquid-phase catalysis compared to gas-phase catalysis could be advantageous for the stability of core–shell catalysts, as high temperatures can lead to restructuring and alloy formation, which is the thermodynamically most stable phase.^18,22^ The selective hydrogenation reaction may proceed differently in the liquid phase due to the presence of solvent molecules, solubility of the reagents and differences in coverage on the nanoparticle surface.^23,24^ For example, the selective hydrogenation of 1-hexyne has been studied with comparable dilute Pd-in-Au alloy nanoparticles in the gas^9^ and liquid^25^ phase. While the results were qualitatively similar, gas-phase operation resulted in a higher selectivity and a lower apparent activation energy for the hydrogenation of 1-hexyne.^9^ In this context, it is relevant to explore the performance of Au–Pd core–shell nanoparticles in liquid-phase selective hydrogenation.

In this work, we study the impact of a core–shell structure on the catalytic performance of bimetallic Au–Pd catalysts in the liquid-phase selective hydrogenation of MBY. Using colloidal synthesis, we prepared well-defined silica supported Au–Pd core–shell and AuPd alloy catalysts with nanoparticles of the same size, composition and weight loading, allowing direct insight in the impact of the metal distribution on their catalytic performance. These nanoparticles have substantially thicker shells (12 atomic Pd layers) compared to the Au–Pd nanorods tested previously in the gas-phase selective hydrogenation of butadiene (1–6 Pd layers),^18^ and their alloying behavior has carefully been characterized through *in* and *ex situ* studies.^22^ We show that controlling the atomic distribution within the Au–Pd nanoparticles is critical in achieving synergy between Pd and Au in the liquid-phase selective alkynol hydrogenation.

## Experimental section

### Chemicals

Trisodium citrate dihydrate (≥99.0%), tannic acid, potassium carbonate (K_2_CO_3_, ≥99.0%), chloroauric acid (HAuCl_4_, 99.9%), sodium tetrachloropalladate(ii) (Na_2_PdCl_4_, 98%), polyvinylpyrrolidone (PVP, Mw 55000), ascorbic acid, potassium bromide (KBr, ≥90%), ascorbic acid (BioXtra, ≥90%) toluene (≥99.9% HPLC grade), 2-methyl-3-butyn-2-ol (MBY, ≥98%) and *n*-octane (≥99%) were obtained from Merck. Aerosil OX 50 was purchased from Degussa. Ultrapure water with a resistivity of 18.2 mΩ cm^−1^ (Millipore Milli-Q grade) was used. All chemicals were used without further purification. The glassware used for AuNP synthesis and Pd overgrowth was cleaned with aqua regia (HCl/HNO_3_ mixture in a 3 : 1 ratio by volume), rinsed thoroughly with Milli-Q water and dried in an oven.

### Synthesis of Au–Pd/SiO_2_ core–shell and alloy catalysts

The colloidal synthesis of the Au–Pd/SiO_2_ core–shell and alloy catalysts was performed as previously reported by our group^22^ and consists of four steps: 1) synthesis of Au nanoparticles of the desired size, 2) Pd overgrowth to form a core–shell structure, 3) deposition onto a SiO_2_ support and 4) oven treatments for ligand removal and alloying, which are further detailed in this section.

#### Au NP synthesis

First, gold nanoparticles (NPs) were synthesized following the procedure of Piella *et al.*^26^ Briefly, an aqueous solution of 450 mL 2.20 mM sodium citrate, 0.30 mL 2.50 mM tannic acid and 3.00 mL 150 mM potassium carbonate were prepared in a 1 L 3-neck round bottom flask. This solution was heated to 70 °C with an oil bath using a condenser and capping the extra necks of the flask. The solution was stirred vigorously throughout the synthesis process. Then, 3.00 mL 25.0 mM HAuCl_4_ were added, while keeping the reaction mixture at 70 °C. Within 3–5 min, the color changed from pale yellow to red, indicating the formation of gold nanoparticles. After 10 minutes, 165 mL of this seed solution were extracted using a pipette and kept apart, and 165 mL of 2.20 mM sodium citrate solution were added to restore the initial reaction volume. When the temperature reached 70 °C again, 1.50 mL 25.0 mM HAuCl_4_ were injected twice with a 10-minute interval. This process was repeated for an additional 8 times, until a total of 30.0 mL 25.0 mM HAuCl_4_ had been added.

#### Pd overgrowth

For the Pd overgrowth, 1.50 mL aqueous polyvinylpyrrolidone solution (1 g/10 mL H_2_O, Mw = 55 000 g mol^−1^) were added to 150 mL of the as-synthesized sodium-citrate-capped gold nanoparticles and stirred overnight in a capped flask at room temperature. Then, the pH was adjusted to 4 with 0.10 M HCl (2.50 mL) to ensure a slow reaction rate during the Pd overgrowth.^27^ Next, 7.80 mL Na_2_PdCl_4_ 10 mM were added to the solution and it was stirred for 5 minutes before rapidly adding 7.80 mL of 40 mM ascorbic acid solution under vigorous stirring to ensure homogeneous reduction of the Pd precursor onto the Au-cores. The solution was left stirring overnight 400 rpm, capped and at room temperature. The resulting Au–Pd core–shell nanoparticles suspensions were centrifuged at 12 000 rcf for 1 h in four 50 mL centrifuge tubes filled with ∼40 mL nanoparticles each, after which the clear supernatant was removed, and the pellet was resuspended in 20 mL ethanol per tube. The nanoparticles were centrifuged again for 1 h at 12 000 rcf and this time the pellet was resuspended in a total of 75 mL of water for storage in the fridge. To avoid the loss of nanoparticles during the washing procedure, it is important that the supernatant is clear after centrifugation.

#### Deposition onto SiO_2_

607 mg of SiO_2_ (Aerosil OX 50) were mixed with 15 mL EtOH and sonicated for 15 minutes to ensure proper dispersion. 40 mL of the Au–Pd core–shell NPs were centrifuged for 1 h at 1200 rcf, redispersed in 15 mL EtOH and added to the silica suspension. The vials were always capped during sonication, which was performed in a Branson 2510 Ultrasonic Cleaner at 40 Hz. After sonicating for approximately one hour, 15 mL of toluene was added to the tube, serving as antisolvent leading to homogeneous deposition of the Au–Pd NPs on the silica support.^18^ Then, it was centrifuged at 2000 rcf for 5 minutes and the clear supernatant was removed. The pellet was dried in an oil bath at 60 °C overnight in the uncapped centrifuge tube in ambient air.

#### Oven treatments

To remove the ligands while keeping the core–shell structure, the supported Au–Pd/SiO_2_ NPs were heated in a U-shaped reactor with a ramp of 5 °C min^−1^ and a flow of 100 mL min^−1^ of 10% O_2_ balanced in N_2_ and were kept at 300 °C for 3 hours. This resulted in a Au–Pd/SiO_2_ core–shell catalyst with 0.98 wt% of metal (0.565 wt% Au and 0.415 wt% Pd) on the Aerosil OX 50 support, as determined by inductively coupled plasma mass-spectrometry (ICP-MS, performed by Mikroanalytisches Laboratorium Kolbe). For part of the sample, an additional heating step was conducted at 450 °C for 1 hour with a ramp of 5 °C min^−1^ and a flow of 100 mL min^−1^ of 10% H_2_ in Ar tubular oven (Thermolyne 79 300 tube furnace), to produce the alloyed AuPd/SiO_2_ catalyst, which was tested immediately after treatment.

#### Monometallic references

The monometallic references were synthesized following the same steps. The Au/SiO_2_ catalyst was synthesized by supporting the Au NPs prior to Pd overgrowth with the same procedure, followed by a 3 hour calcination at 300 °C with of 100 mL min^−1^ of 10% O_2_ balanced in N_2_, resulting in a 0.93 wt% catalyst as determined by ICP. On the other hand, the Pd/SiO_2_ catalyst was synthesized by supporting ∼20 nm Pd nanocubes and calcine them for 12 h at 450 °C with a ramp of 5 °C min^−1^ and a flow of 200 mL min^−1^ of 20% O_2_ balanced in N_2_. The Pd nanocubes were synthesized by heating 205 g of PVP, 125 mg of ascorbic acid, 1.223 g of KBr and 16.0 mL of deionized water to 80 °C and after 10 min of stirring, introducing 113.2 mg of Na_2_PdCl_4_ in 6.0 mL of water and maintaining the solution at 80 °C for 3 h.^28^ The resulting Pd/SiO_2_ catalyst consisted of ∼25 nm Pd nanoparticles with a 0.88 wt% metal loading.

### Characterization

**Transmission electron microscopy** (TEM) was performed on a Talos F200x (Thermo Fisher Scientific) operated at 200 kV. Particle size distributions of the free-standing nanoparticles were obtained by measuring 100 to 200 nanoparticles from bright field TEM images. First, a threshold was applied to mask the nanoparticles, and the size was analyzed using the analyze particles function in ImageJ.^29^ Size distributions for the supported nanoparticles were obtained by manually measuring 50–100 nanoparticles. For energy-dispersive X-ray (EDX) mapping, the microscope was operated in scanning-TEM (STEM) mode, with a pixel size of 0.3827 nm and a dwell time of 5 μs. The EDX maps of the free-standing Au–Pd core–shell nanoparticles were acquired with 512 × 512 pixels and a screen current of 1.6 nA with a total time of ∼10 minutes. The EDX maps of the supported nanoparticles were acquired with less electron dose (0.7 nA screen current), at the same magnification but with smaller field of view to avoid charging of the silica support. The Super-XTM EDX detector present in the microscope was used to collect the EDX signal. The EDX signal was quantified using the Velox software. The EDX maps were prefiltered by averaging 5 pixels before quantification. Both Au and Pd were quantified using the L-lines.

**N**_**2**_ **physisorption** isotherms were measured at −196 °C on a Micromeritics TriStar 3000 apparatus after drying overnight at 200 °C under vacuum. The specific surface area was calculated using the BET equation (0.05 < *p*/*p*_0_ < 0.25).

### Catalytic testing

The catalytic properties of the Au–Pd/SiO_2_ catalysts were tested in the liquid-phase hydrogenation of 2-methyl-3-butyn-w-ol (MBY) to 2-methyl-3-buten-2-ol (MBE) in a batch reactor. Typically, ∼15 mg of catalyst, consisting of 6.1–6.4 × 10^−8^ moles of surface atoms for the core–shell, alloy and Au catalyst and ∼8.4 × 10^−8^ moles of surface atoms for the Pd catalyst, 3.00 mL MBY, 0.20 mL *n*-octane and 100 mL toluene were added to the reactor. The reactor was a 300 mL stainless steel Parr Instrument Company autoclave equipped with a glass liner, a gas inlet port, a mechanical stirrer and a liquid-only sampling line to sample ∼2 mL. Once the reactor was loaded and sealed, the head space of the reactor was purged 3 times with N_2_, pressurized with 30 bar N_2_ and heated to 50 °C while stirring at 400 rpm. Once the temperature was stable, the reactor outlet was flushed three times and the atmosphere was switched to 30 bar H_2_ after flushing three times with H_2_. Immediately after pressurizing at 30 bar H_2_ the stirring speed was increased to 800 rpm and the sample at *t* = 0 was taken. During the reaction, 1 mL aliquots were taken at regular time intervals (the outlet was always flushed once before taking an aliquot for analysis), filtered with 0.45 μm PTFE filters and analyzed with a Bruker 430 gas chromatograph using an Agilent VF-5 ms 15 m fused silica column with an internal diameter of 0.25 mm, a film thickness of 0.25 μm and a cage size of 7 inch and a FID detector. Afterwards, the reaction mixture was filtered for further analysis of the recovered catalyst. A blank test was performed before every test with catalyst to ensure proper cleaning of the reactor and conversion levels <5% over 1 h. The relative concentrations of MBY, MBE and MBA were calculated from the relative area of the peaks of this compounds, assuming a 100% carbon balance, which was calculated as described in Fig. S1. The selectivity to MBE was calculated by dividing the MBE% by the sum of MBE and MBA%.

### Turnover frequency (TOF) calculation

The turnover frequency (TOF) was determined by assuming first order kinetics. The experimental data was fitted for the integrated rate equations for [MBY]_*t*_, [MBE]_*t*_, and [MBA]_*t*_, using Python to obtain the reaction constants *k*_1_ and *k*_2_ for the hydrogenation of MBY and MBE, respectively.^30,31^ We assumed unidirectional reactions (
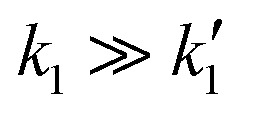
 and 
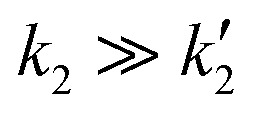
) and a constant reaction volume. Since [MBY]_0_, k_1_ and k_2_ are fitted independently, after the first fit we allowed a 20% variation of the k_1_ parameter. No boundaries were applied for the calculation of k_2_ after the MBE fit to ensure proper fitting in the MBA curve. The fits usually had a *R*^2^ > 0.9. We introduced one minute of induction time to allow proper mixing of the reagents. The final *k*_1_ and *k*_2_ constants used for TOF calculations are an average of the fitting parameters, and the error corresponds to the standard deviation between this values.[MBY]_*t*_ = [MBY]_0_ × e^−*k*_1_*t*^
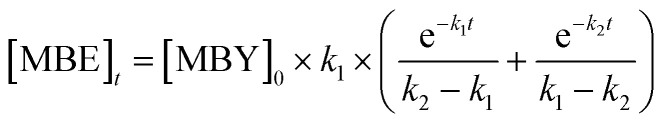

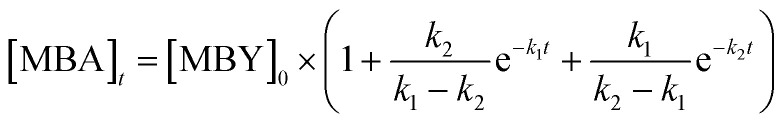
TOFs were expressed in number of converted MBY molecules per second per metal surface atom, calculated as follows.
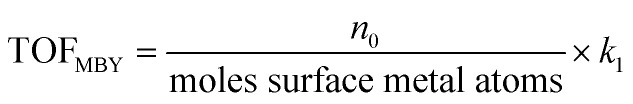

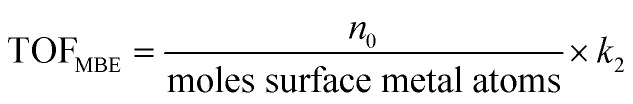
where *n*_0_ is the initial amount of MBY in moles and *k*_1_ and *k*_2_ are the reaction constants in s^−1^ obtained from fitting the previous first order kinetics equations.

The total moles of surface metal atoms loaded in the reactor were calculated by multiplying the moles of surface atoms in an individual nanoparticle by the number of nanoparticles loaded. This calculation yielded an effective TOF, averaging all surface sites that may not be equally active or accessible.

The number of surface atoms (in moles) per nanoparticle was calculated by using the nanoparticle size (*d*_NP_) obtained from TEM to calculate the surface area and the Pd lattice parameter (*a* = 0.389 nm) to calculate the atomic surface density of the 111 facet,^32^ which for Pd is 15.3 Pd atoms* nm^−2^,^33^ and the Avogadro number *N*_A_:
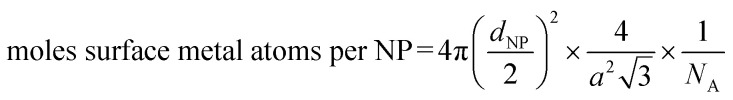
The total number of nanoparticles was calculated by dividing the mass of loaded metal, calculated with the sample weight loaded into the reactor and the weight loading, by the mass of a single nanoparticle.
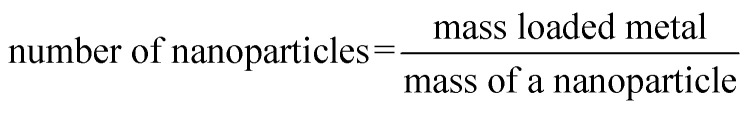
where the mass of a nanoparticle is calculated by using the volume of the nanoparticle calculated with the diameter from TEM (*d*_NP_), and the nanoparticle density, calculated with a weighted average of the Au and Pd present in each nanoparticle:NP_Mass_ = NP_volume_ × NP_density_
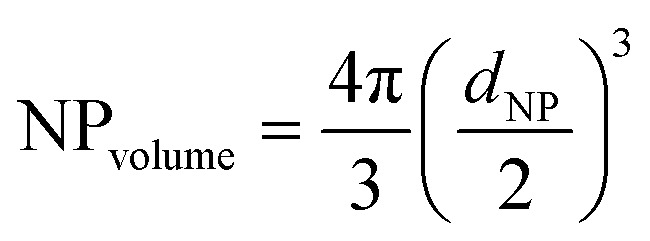
NP_density_ = *ρ*_Au_ × *X*_Au_ + *ρ*_Pd_ × *X*_Pd_where *X*_Au_ and *X*_Pd_ are the atomic fraction of Au and Pd and *ρ*_Au_ and *ρ*_Pd_ are the Au and Pd density (19.3 × 10^−21^ and 12.0 × 10^−21^ g* nm^−3^).

## Results

### Synthesis and characterization of Au–Pd core–shell and alloy catalysts

**Fig. 1:**
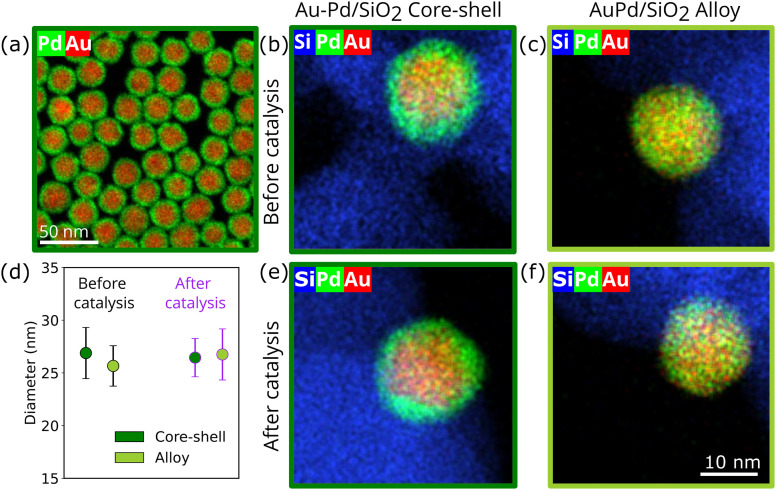
Electron microscopy characterization of the Au–Pd/SiO_2_ core–shell and alloy nanoparticles. STEM-EDX maps of the (a) as-synthesized free-standing Au–Pd core–shell nanoparticles, (b) supported Au–Pd/SiO_2_ core–shell catalyst after ligand removal, and (c) supported AuPd/SiO_2_ alloy catalyst. (d) Nanoparticle size distribution of the core–shell and alloy catalysts before (grey outline) and after (purple outline) catalysis, obtained from TEM images of the supported nanoparticles, counting >50 nanoparticles per sample. (e) STEM-EDX map of the used Au–Pd/SiO_2_ core–shell catalyst. (f) STEM-EDX map of the used AuPd/SiO_2_ alloy catalyst. All EDX maps show Au in red, Pd in green and Si in blue, and b, d, e and f share the same scale bar shown in panel f.

Au–Pd core–shell nanoparticles (NPs) uniform in size, shape and composition were synthesized by colloidal synthesis.^22^ The core–shell structure of the NPs is clearly visible in the scanning transmission electron microscopy energy-dispersive X-ray (EDX-STEM) map in Fig. 1a, where the Pd signal extends further than the Au signal, indicating that only Pd is present at the surface of the core–shell nanoparticles. EDX revealed a composition of 58 ± 4 atomic% Pd, uniform between nanoparticles (Fig. S2), matching the 57.7 atomic% Pd obtained from bulk analysis with inductively coupled plasma (ICP). Transmission electron microscopy (TEM) images showed an average diameter of 24.7 ± 2.4 nm (Fig. S3). To ensure that particle size effects did not influence the catalytic performance, a >20 nm particle size was deliberately chosen, as smaller nanoparticles can show a size-dependent effect.^34,35^ The core–shell Au–Pd nanoparticles were deposited on a commercial silica support (Aerosil OX 50, 50 m^2^ g^−1^) and the polyvinylpyrrolidone ligands were removed by calcination.^22^Fig. 1b shows an EDX map of a supported nanoparticle after this calcination treatment, showing that the Au–Pd core–shell structure remained unaffected after the heat treatment. Part of the batch of supported core–shell particles was alloyed during an additional H_2_ treatment at 450 °C. The EDX map of the resulting AuPd/SiO_2_ alloy catalyst is shown in Fig. 1c, showing full Au and Pd mixing in a single nanoparticle. A schematic of the full synthesis process is shown in Fig. S4. The resulting Au–Pd/SiO_2_ core–shell and AuPd/SiO_2_ alloy catalysts had a metal weight loading of 1.0 wt%, as determined by ICP, and the nanoparticles were well-dispersed on the silica support, as shown in Fig. S5. As the Au–Pd NPs of the core–shell and alloy catalysts were produced in the same batch, they had exactly the same composition and size, which are variables that need to be kept constant as they can affect the catalytic performance.^14,34,35^ Therefore, these samples allowed the direct evaluation of the impact of the atomic distribution on the performance of Au–Pd catalyst in liquid-phase selective hydrogenation catalysis.

### Effect of the atomic distribution in the liquid-phase selective hydrogenation of MBY

**Fig. 2:**
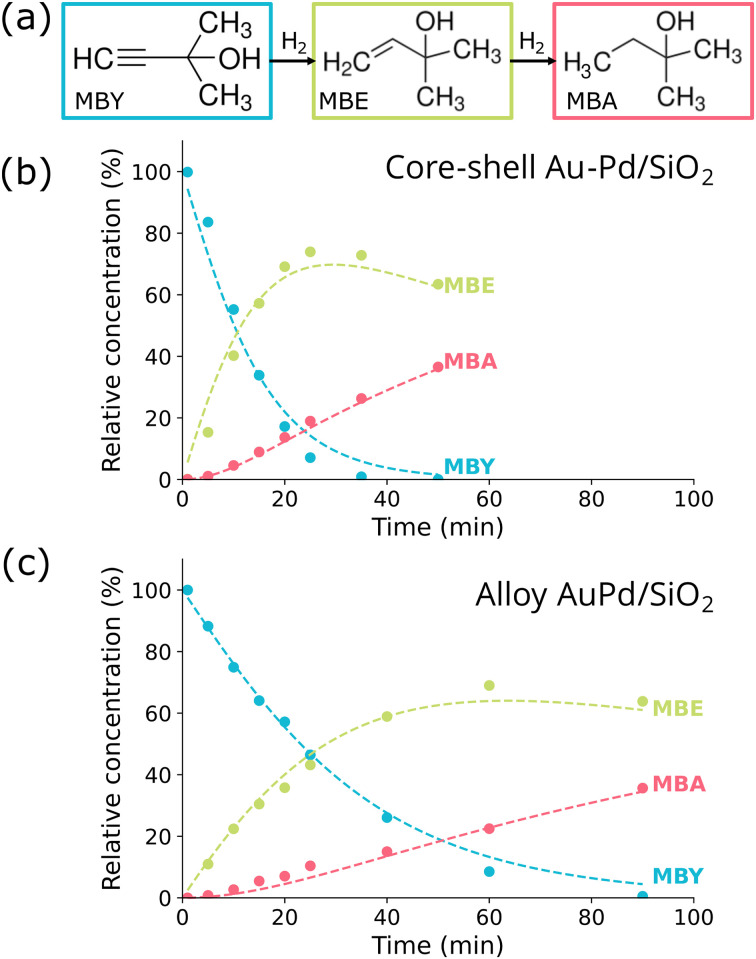
Concentration evolution over time for the core–shell and alloy catalysts in the selective hydrogenation of MBY. (a) Reaction scheme of the selective hydrogenation of MBY (2-methyl-3-butyn-2-ol) to MBE (2-methyl-3-buten-2-ol, desired product) and MBA (2-methylbutan-2-ol, undesired product). (b) Reaction profile of the Au–Pd/SiO_2_ core–shell catalyst and (c) of the AuPd/SiO_2_ alloy catalyst. The blue, green and red datapoints correspond to the relative concentration of MBY, MBE and MBA, respectively, at each point in time. The dashed lines show the fits of the data using the first order kinetics equations as described in the experimental section. The reaction conditions were ∼15 mg catalyst, 0.3 mL MBY, 0.2 mL octadecane, 100 mL toluene, 30 bar H_2_, 50 °C reaction temperature and 800 rpm stirring.

The core–shell and alloy Au–Pd catalysts were tested for the selective hydrogenation of 2-methyl-3-butyn-2-ol (MBY) in the liquid-phase in a batch reactor. As shown in the Fig. 2a, MBY is hydrogenated into the desired product MBE (2-methyl-3-buten-2-ol), which can undergo a second hydrogenation step to form MBA (2-methylbutan-2-ol), the undesired product. The liquid-phase reaction was performed in an autoclave with toluene as a solvent, under vigorous stirring and a pressure of 30 bar H_2_.^6^ Aliquots were taken at different time stamps and the products were analyzed with gas chromatography (see experimental section). Both catalysts were active and MBY was fully converted within 30–90 minutes of reaction. TEM analysis of the catalyst after reaction showed no visible signs of nanoparticle growth (Fig. S5), with no significant changes in the size distribution (Fig. 1d). STEM-EDX maps showed that the core–shell and alloy atomic distribution remained unaffected by the catalytic reaction (Fig. 1e and f).

Fig. 2b and c show the relative concentration profiles of MBY, MBE and MBA as function of reaction time for the core–shell and alloy catalyst, respectively. The conversion of MBY was clearly faster for the core–shell catalyst: 95% MBY conversion was reached after 36 minutes for the core–shell catalyst (Fig. 2b and S6a) *versus* 77 minutes for the alloy catalyst (Fig. 2c and S6b) on average. In both cases, as MBY was converted, MBE and MBA were formed. To quantitatively analyze the reaction and compare the catalysts, we fitted the reaction profile with first order reaction kinetics,^30^ as shown by the dashed lines in Fig. 2. The resulting turnover frequencies (TOFs) normalized per surface metal atom are summarized in Table 1. There were no discernible effects of varying the amount of loaded catalyst on the computed TOF, suggesting that our reactor had no mass-transfer limitations (Table S1). Internal mass transfer limitations are unlikely due to the large pore volume of the support and the low nanoparticle loading. Indeed, the BET surface area and pore volume for the empty support and nanoparticle catalyst are identical (Fig. S7 and Table S2), confirming that the presence of the particles did not lead to any pore blocking. The catalyst met the Weisz–Prater criteria, indicating no internal mass transfer limitations (Note S1). Moreover, the carbon balance was close to 100%, indicating no side-products were formed (Fig. S1). The TOF of MBY conversion was ∼2× higher when comparing the core–shell to the alloy, and ∼8× and ∼4× times higher than the TOF of the undesired MBE conversion, for the core–shell and the alloy, respectively. When the TOFs are normalized per Pd surface atom, assuming full Pd surface coverage in the core–shell Au–Pd/SiO_2_ and a homogeneous alloy composition (58 atomic% Pd, 42 atomic% Au) at the surface for alloyed AuPd/SiO_2_, the alloy shows similar TOFs for both MBY and MBE conversion (Fig. S8 and Table S3). However, it should be noted that it is difficult to assess the exact surface composition for the alloyed nanoparticles during liquid-phase hydrogenation, and that Au surface segregation is probable upon H_2_ exposure.^22,36^ The core–shell catalyst showed a 80 ± 2% selectivity to MBE at 91% MBY conversion while this was somewhat lower for the alloyed catalyst (74 ± 2% MBE selectivity). Thus, the core–shell catalyst clearly outperformed the alloy catalyst both in catalytic activity as well as selectivity towards the desired MBE product.

**Table 1:** Summary of the turnover frequencies (TOF) of the investigated bimetallic and monometallic catalysts, obtained by kinetic fitting of the full reaction profile (see experimental section)

Catalyst	TOF MBY conversion (mol MBY * mol surface metal atoms^−1^ * s^−1^)	TOF MBE conversion (mol MBE * mol surface metal atoms^−1^ * s^−1^)
Core–shell AuPd/SiO_2_	653 ± 66	84 ± 10
Alloy AuPd/SiO_2_	322 ± 39	75 ± 37
Monometallic Au/SiO_2_	59 ± 5	7 ± 3
Monometallic Pd/SiO_2_	168 ± 17	27 ± 10

### Comparison to monometallic catalysts

We compared the Au–Pd core–shell and alloy catalysts to Au and Pd monometallic references. Fig. S9 shows TEM images of these catalysts before and after catalysis, together with the size distributions. The monometallic Au/SiO_2_ reference consisted of silica supported ∼20 nm Au nanoparticles synthesized prior to Pd overgrowth and were monodisperse (20.2 ± 1.9 nm). The Pd reference was obtained by calcining a sample consisting of ∼20 nm Pd nanocubes on silica, leading to spherical nanoparticles with a broader size distribution (25.4 ± 4.6 nm). The catalysts were tested for the selective hydrogenation of MBY by loading comparable metal surface atoms. The experimental reaction profiles, including kinetic fitting, are shown in Fig. S10. While the Pd catalyst reached 95% MBY conversion at 105 min, the Au catalyst only reached 95% conversion after 6.4 h, showing a much lower catalytic activity. Thus, both Au–Pd core–shell and alloyed catalysts are more active than monometallic Pd.

**Fig. 3:**
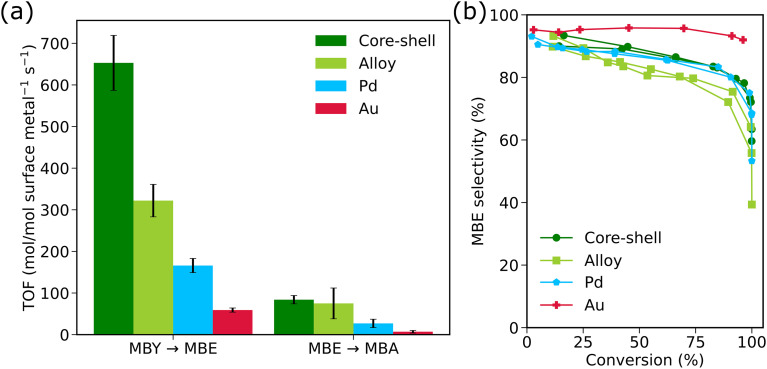
Comparison of the catalytic performance of the Au–Pd/SiO_2_ core–shell and AuPd/SiO_2_ alloy catalysts with the monometallic Pd/SiO_2_ and Au/SiO_2_ catalyst in the selective hydrogenation of MBY (a) turnover frequency (TOF) comparison for the MBY to MBE and the MBE to MBA conversions. The TOF is expressed as mol MBY or MBE converted for each mole of surface metal per second. The error bars represent the largest source of uncertainty, either the standard deviation from two independent turnover frequency (TOF) measurements or the fitting error. (b) MBE selectivity *vs.* MBY conversion plot. The core–shell catalyst is shown in dark green, the alloy in light green, Pd in blue and Au in red. The reaction conditions were ∼15 mg loaded catalyst, with 6.1–6.4 × 10^−8^ moles of surface atoms for the core–shell, alloy and Au catalyst and ∼8.4 × 10^−8^ moles of surface atoms for the Pd catalyst, 30 bar H_2_, 100 mL toluene, 3 mL MBY, stirring at 800 rpm.

Fig. 3 shows a comparison of all tested catalysts. The TOFs, expressed as mol converted per mole of surface metal are summarized in Table 1 and plotted in Fig. 3a. In the MBY to MBE conversion, the Au–Pd core–shell is the most active catalyst, followed by the AuPd alloy, the Pd and lastly the Au catalyst. The core–shell and alloy catalysts show similar TOFs for the MBE to MBA conversion, which are ∼3× higher than for the Pd catalysts and ∼11× higher than for the Au catalyst. This gives rise to the Pd catalyst showing similar selectivity as the core–shell catalyst and the Au catalyst showing a >90% selectivity over the full conversion range (Fig. 3b). Surprisingly, the selectivity of the AuPd alloy catalyst is lower than the catalysts with a fully Pd covered surface. In terms of combined activity and selectivity, the Au–Pd core–shell catalyst is the best performing catalyst.

## Discussion

The decreased selectivity in the alloyed catalyst compared to the core–shell and monometallic Pd catalyst could be ascribed to differences in the binding site and (relative) binding strength of MBY and MBE on the catalyst surface. Density functional theory (DFT) has shown that MBY binding involves multiple Pd atoms and therefore preferentially binds to Pd atoms located at the terrace sites in the nanoparticle.^11,37^ MBE, on the other hand, binds to single Pd atoms, which are typically present at the undercoordinated edge and corner sites in Pd nanoparticles.^11,37^ This gives rise to a nanoparticle shape and size dependent selectivity, with higher selectivities for large and/or more cubical Pd nanoparticle structures that contain a relatively high fraction of flat terrace sites compared to Pd octahedra and/or small nanoparticles that exhibit more undercoordinated sites.^34,35^ Thus, the lower selectivity of the Au–Pd alloy catalyst compared to the Au–Pd core–shell catalyst could be due to enhanced preferential binding of MBY over MBE on the fully covered Pd surface of the Au–Pd core–shell nanoparticles compared to the more dilute Pd-in-Au surface of the Au–Pd alloyed catalyst, giving rise to a clear difference in TOF for MBY (653 *vs.* 322 s^−1^). At the same time, the hydrogenation of MBE is similar for both catalysts as is evident from the comparable TOFs for MBE conversion (84 *vs.* 75 s^−1^). This is in line with a theoretical study on PdZn nanoparticles that concluded that when doping a Pd surface with Zn the binding strength of MBY decreases, while the binding energy of MBE remains the same. This could explain why the TOF for MBY decreases for the AuPd alloy compared to the core–shell, whereas the TOF for MBE remains unaffected.^5^

We found that the Au–Pd core–shell catalyst exhibited a strongly enhanced catalytic activity compared to the AuPd alloy and monometallic Pd and Au catalysts while maintaining high selectivity to MBE also at high MBY conversion levels. Strain effects have previously been invoked to explain the enhanced activity of Au–Pd core–shell structures in the gas-phase selective hydrogenation of butadiene^18^ and the liquid-phase selective oxidation of benzyl alcohol^19,21^ (∼50× and ∼3.5×, respectively). This is because tensile strain can increase the binding energy of reagents.^18^ In our work, the core–shell structures show a ∼2× and ∼4× increase in TOF compared to the alloy and monometallic Pd catalysts, respectively. Strain effects arise from the lattice mismatch between Au (408 pm) and Pd (389 pm), leading to a tensile strained Pd lattice in the shell of the Au-core Pd-shell nanoparticles. This lattice strain can persist up to 30 Pd layers before the Pd lattice returns to the bulk value,^38^ and is known to substantially alter the binding strength of hydrogen and hydrocarbons.^18^ Contrarily, additional electronic effects induced by the differences in electronegativity of the core and shell metal tend to be negligible for shell layers thicker than 1–2 atomic layers.^21,39^ In our Au-core Pd-shell nanoparticles, the Pd-shell is relatively thick (10–12 atomic layers) and it is grown epitaxially,^22^ meaning that it follows the crystal lattice of the underlying Au-core and therefore is likely strained. Moreover, since the Pd-shell consists of 12 Pd atomic monolayers, no Au impurities are expected at the surface. Thus, it is expected that in our core–shell system strain effects dominate the catalytic behavior, which together with a full Pd surface provide enhanced activity and selectivity in Au–Pd core–shell nanoparticles.

## Conclusions

In conclusion, we showed that synergy between Au and Pd in the liquid-phase hydrogenation of MBY is only achieved in a core–shell design with the Pd atoms located in the shell and Au residing in the core. Our approach, relying on controlled colloidal synthesis of well-defined silica supported Au–Pd core–shell and alloy catalysts, allowed direct evaluation of the catalytic performance as a function of metal distribution, probed under identical conditions. Our core–shell catalysts combined high activity, good selectivity and high stability, and clearly outperformed its monometallic and alloy counterparts. These favorable catalytic properties likely arise from lattice strain in the Pd-shell caused by the lattice mismatch between the Au-core and Pd-shell. Altogether, our study demonstrates that core–shell nanoparticle catalysts are promising structures for liquid-phase selective hydrogenation catalysis, exhibiting catalytic behavior distinctly different from to the conventional alloyed and monometallic nanoparticle catalysts.

## Conflicts of interest

The authors have no conflicts of interest to declare.

## Supplementary Material

CY-015-D5CY00889A-s001

## Data Availability

The data supporting this article have been included as part of the supplementary information (SI). Supplementary information is available. See DOI: https://doi.org/10.1039/d5cy00889a.
