# Supplementary material for: Au–Pd core–shell nanoparticles for enhanced catalytic performance in liquid-phase selective hydrogenation
Source: Catal Sci Technol. 2025 Oct 14;15(22):6757–65. doi: 10.1039/d5cy00889a (PMC12520061; doi:10.1039/d5cy00889a)
Supplement: CY-015-D5CY00889A-s001 [file CY-015-D5CY00889A-s001.pdf]

Supplementary Information (SI) for Catalysis Science & Technology.
This journal is © The Royal Society of Chemistry 2025

Supporting information:

Au-Pd core-shell nanoparticles for enhanced catalytic performance in liquid-phase selective hydrogenation

Marta Perxés Perich[1], Kristiaan H. Helfferich[1], Petra E. de Jongh[1] and Jessi E.S. van der Hoeven[1]
* j.e.s.vanderhoeven@uu.nl
[1] *Materials Chemistry and Catalysis, Debye Institute for Nanomaterials Science, Utrecht University, 3584 CG Utrecht, The Netherlands*

Table of contents

Supplementary Figures 1-10 2
Supplementary tables 1-3 7
Note S1: Calculation of the Weisz-Prater criteria 8
References 8

Supplementary Figures 1-10

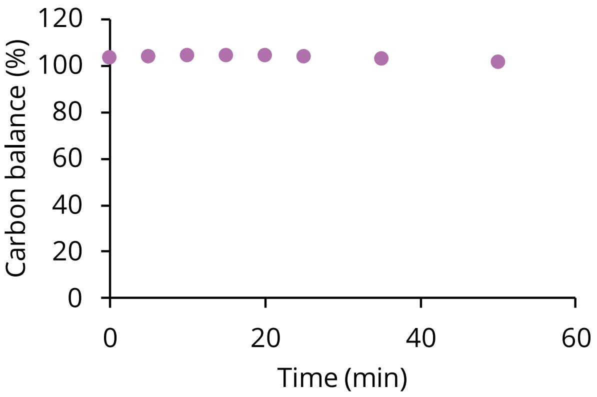

Figure S1. Carbon balance during the selective hydrogenation of MBY over Au-Pd/SiO_2 core-shell catalyst. All samples showed the same evolution of the carbon balance. As no internal standard was used in the reaction, a calibration series with known concentrations of MBY, MBE and MBA was performed, and the response factors RF_{MBY}, RF_{MBE} and RF_{MBA} of the relative peak areas were calculated. The carbon balance was then calculated as an addition of the relative concentration at each time point multiplied by the response factor:

$$Carbon\ balance = 100 * \left(RF_{MBY} * \frac{A_{MBY}}{A_{MBY}+A_{MBE}+A_{MBA}} + F_{MBEs} * \frac{A_{MBE}}{A_{MBY}+A_{MBE}+A_{MBA}} + RF_{MBA} * \frac{A_{MBA}}{A_{MBY}+A_{MBE}+A_{MBA}}\right)$$

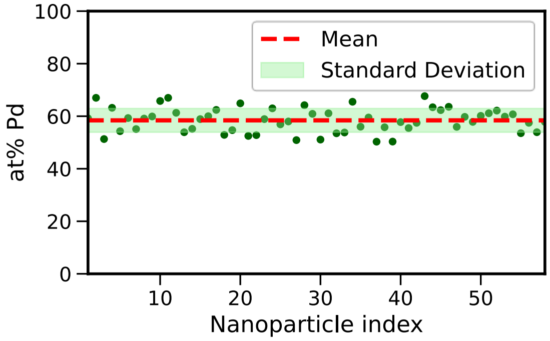

Figure S2. The Pd atomic % is uniform between nanoparticles. Atomic % of Pd obtained from EDX maps of individual nanoparticles, represented in each green dot. The red dashed line represents the mean and the green shadow the standard deviation (58 ± 5 at% Pd).

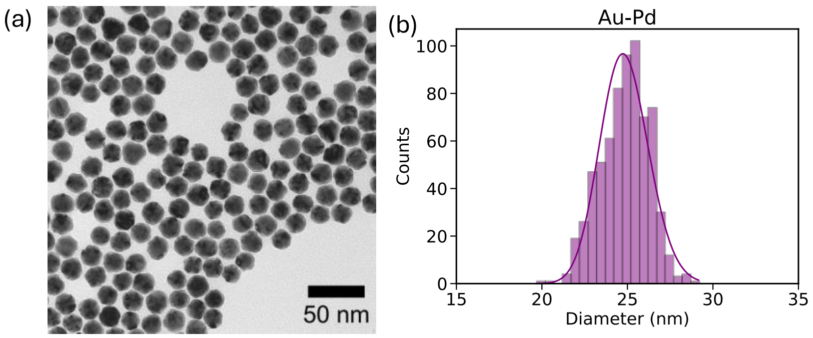

Figure S3. Size distribution of unsupported Au-Pd nanoparticles (a) Transmission electron microscopy (TEM) image of as-synthesized free-standing Au-Pd core-shell nanoparticles. (b) Size distribution of the Au-Pd nanoparticles obtained by measuring more ~600 nanoparticles. Note that the supported alloyed AuPd nanoparticles have the same nanoparticle size as they originate from the same catalyst.

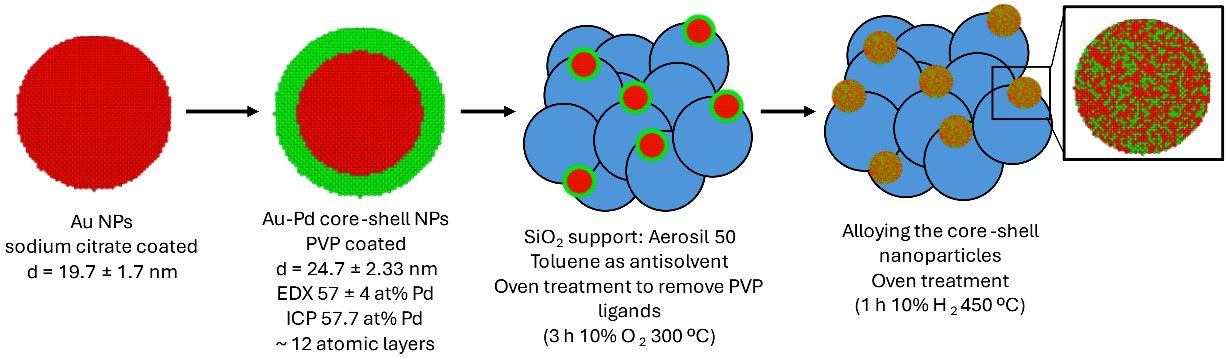

Figure S4. Schematic representation of the synthesis of the Au-Pd/SiO_2 core-shell and alloy catalysts.

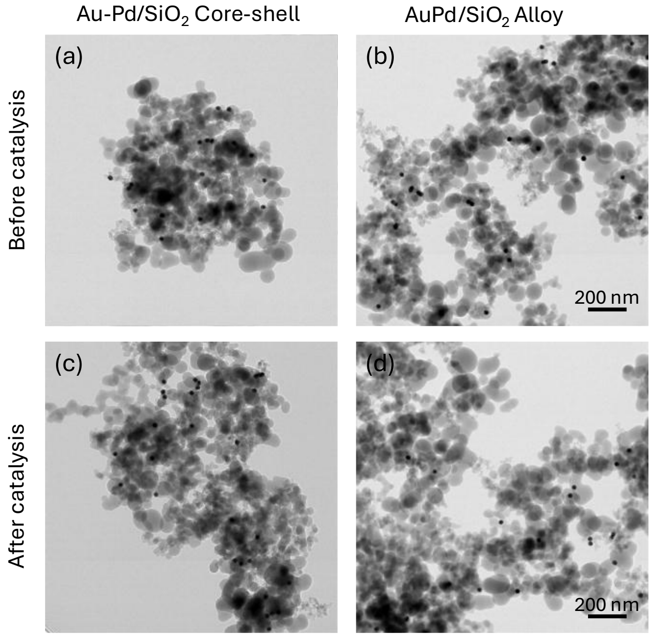

Figure S5. Nanoparticle growth is not observed after the heat treatments to form the alloy nanoparticles nor after catalysis. Representative TEM image of (a) fresh and (c) used Au-Pd/SiO_2 core-shell and fresh (b) and used (d) AuPd alloy catalysts.

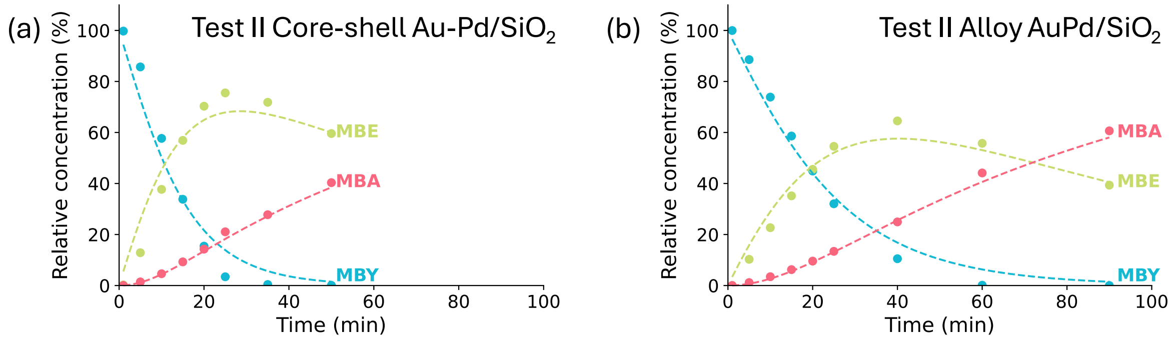

Figure S6. Reaction profile for the core-shell and alloy catalysts in the selective hydrogenation of MBY, test II. (a) Reaction profile of the Au-Pd/SiO_2 core-shell catalyst and the (b) AuPd/SiO_2 alloy catalyst. The dotted lines correspond to the fitting of the data using the first order kinetics equations as described in the experimental sections. The blue, green and red datapoints correspond to the relative concentration of MBY, MBE and MBA, respectively at each time point. The dotted lines show the fits of the data using the first order kinetics equations as described in the experimental section. The reaction conditions were ~15 mg catalyst, 0.3 mL MBY, 0.2 mL octadecane, 100 mL toluene, 30 bar H_2, 50 °C reaction temperature and 800 rpm stirring.

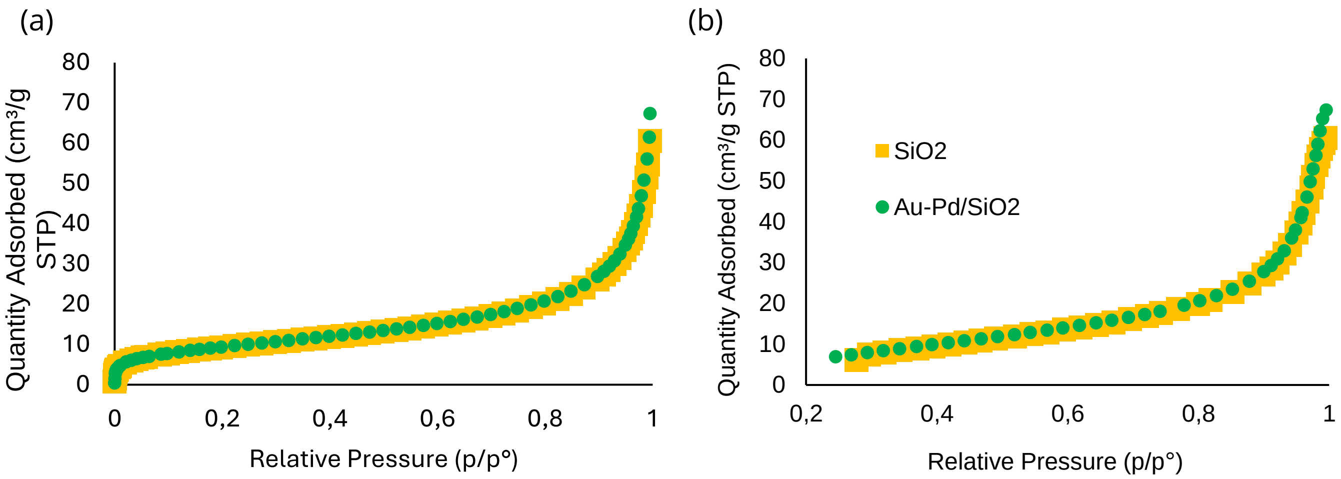

Figure S7. Nitrogen physisorption isotherms of the SiO_2 empty support and the core-shell Au-Pd/SiO_2 catalyst. (a) Adsorption branch and (b) desorption branch.

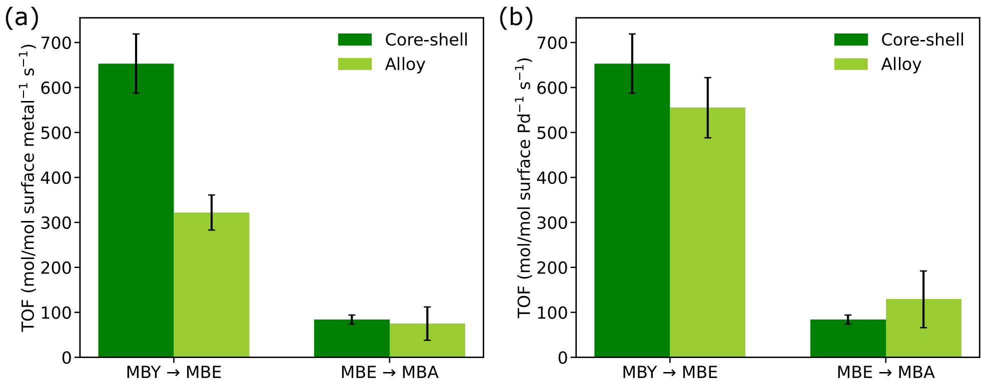

Figure S8. TOFs for the core-shell and alloy catalysts (a) normalized per surface atom (Au+Pd) (b) normalized per Pd surface atom. For the core-shell Au-Pd/SiO_2 full Pd surface coverage was assumed. For the alloy AuPd/SiO_2 we assumed a randomly mixed Au and Pd surface with no surface segregation, thus a 58% of Pd surface atoms, according to the nanoparticle composition obtained with EDX and ICP.

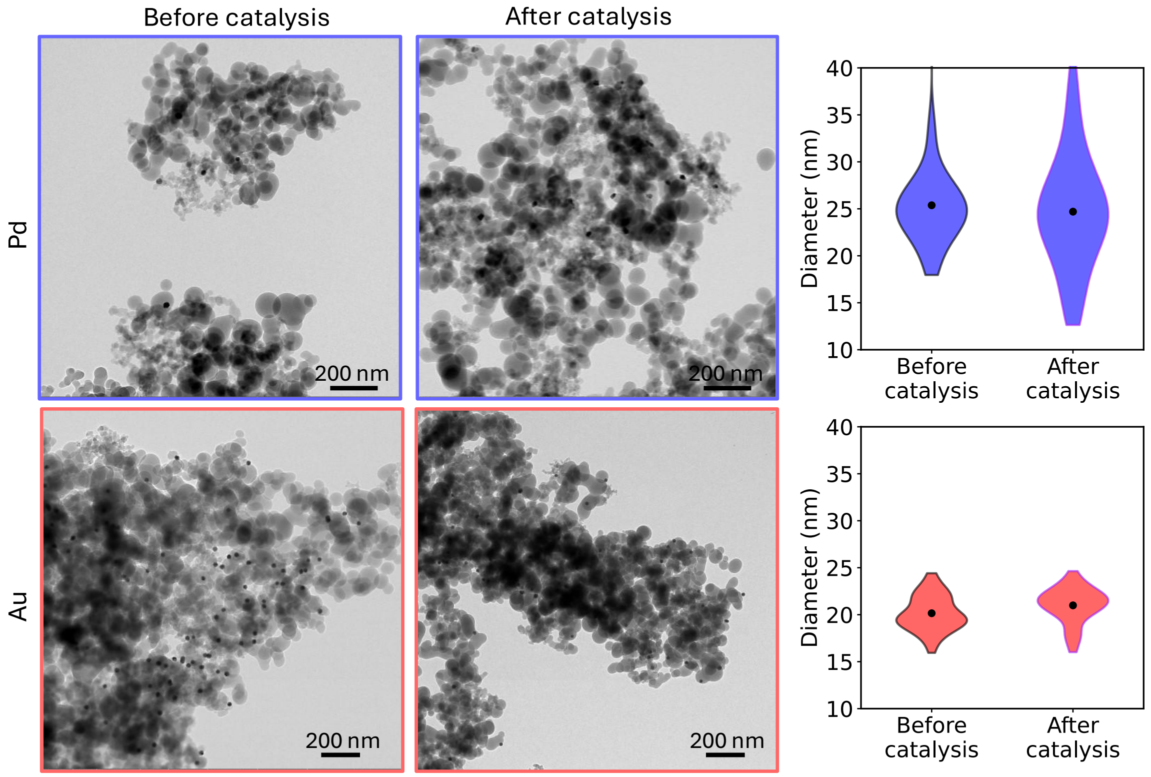

Figure S9. TEM characterization of the monometallic Pd/SiO_2 (blue) and Au/SiO_2 (red) monometallic catalysts. TEM images of each catalyst before and after catalysis, together with the measured size distribution. The average particle diameters were 25.4 ± 4.6 and 24.7 ± 6.3 nm for the Pd/SiO_2 catalyst before and after catalysis, respectively, and 20.2 ± 1.9 and 20.9 ± 1.9 nm for the Au/SiO_2 catalyst before and after catalysis, respectively.

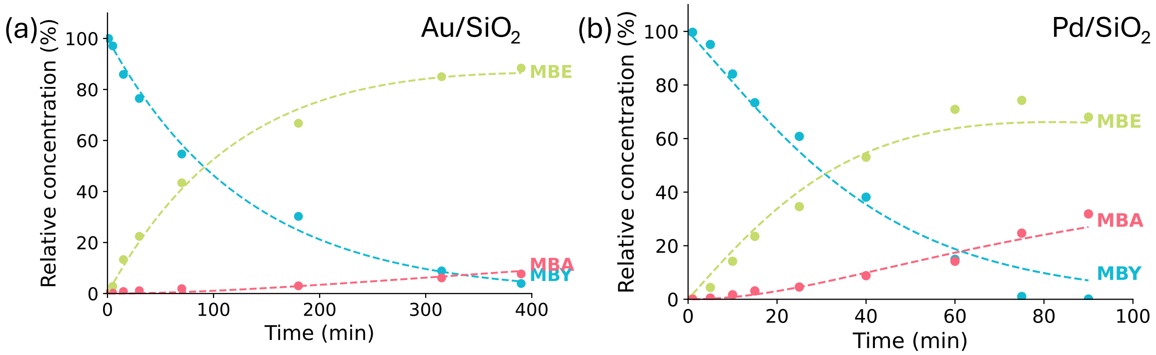

Figure S10. Reaction profiles of the monometallic Au/SiO_2 (a) and Pd/SiO_2 (b) catalysts in the selective hydrogenation of MBY.. The dotted lines correspond to the fitting of the data using the first order kinetics equations as described in the experimental section. The blue, green and red datapoints correspond to the relative concentration of MBY, MBE and MBA, respectively at each time point. The reaction conditions were ~15 mg catalyst, with $6.4 \cdot 10^{-8}$ moles of surface atoms Au catalyst and ~$8.4 \cdot 10^{-8}$ moles of surface atoms for the Pd catalyst, 0.3 mL MBY, 0.2 mL octadecane, 100 mL toluene, 30 bar H_2, 50 °C and 800 rpm stirring.

Supplementary tables 1-3

Table S1. Turn-over-frequency (TOF) comparison of Au-Pd core-shell catalysts with different amount of catalyst loaded per test.

Catalyst	TOF MBY (mol MBY * mol surface metal^{-1} *s^{-1})	TOF MBE (mol MBY * mol surface metal^{-1} * s^{-1})
Core-shell Au-Pd/SiO_2	654 ± 66	81 ± 10
Repeat test	652 ± 66	86 ± 10
1/3 x loaded catalyst (5 mg)	795 ± 77	92 ± 77
2x loaded catalyst (30 mg)	630 ± 74	134 ± 11 *

The errors correspond to the error of the fitted parameters: three different curves corresponding to MBY, MBE and MBA are fitted to the experimental points, and a k_1 and k_2 are obtained in each individual fit. The standard deviation between those observed k_1 and k_2 are used to calculate the error of the final TOF.

** In the test with 30 mg of loaded catalyst full MBY conversion was reached in the first 15 minutes, so less datapoints were available for fitting. In particular, the MBE fit had a r^2~0.85, lower than the r^2>0.90 the other curves had, which could have caused the variation of the calculated TOF.*

Table S2. BET surface area and pore volume of the empty SiO_2 support material and the core-shell Au-Pd/SiO_2 catalyst as determined by N_2 physisorption.

Sample	BET Surface Area	Pore volume
SiO_2 empty support	33.9 m^2/g SiO_2	0.09 cm^3/g SiO_2
Au-Pd/SiO_2	34.0 m^2/g SiO_2	0.10 cm^3/g SiO_2

Table S3. Summary of the turnover frequencies (TOF) of the investigated bimetallic- and monometallic catalysts, obtained by kinetic fitting of the full reaction profile (see experimental section), normalized per Pd surface atom*.

Catalyst	TOF MBY conversion (mol MBY * mol Pd surface atoms^{-1} *s^{-1})	TOF MBE conversion (mol MBE * mol Pd surface atoms^{-1} * s^{-1})
Core-shell AuPd/SiO_2	653 ± 66	84 ± 10
Alloy AuPd/SiO_2	555 ± 67	129 ± 63

* For the core-shell Au-Pd/SiO_2 full Pd surface coverage was assumed. For the alloy AuPd/SiO_2 we assumed a randomly mixed Au and Pd surface with no surface segregation, thus a 58% of Pd surface atoms, according to the nanoparticle composition obtained with EDX and ICP.

Note S1: Calculation of the Weisz-Prater criteria

The Weisz-Prater criterium to rule out the presence of internal mass-transfer limitations was estimated as

$$\frac{d^2 \rho R_{MBY}}{6 D_{e,MBY} C_{MBY}} \left(\frac{n+1}{2}\right) < 0.08$$

Where d^2 is the is the catalyst particle diameter (m), ρ is the density of the catalyst ($kg{\cdot}m^{-3}$), R_{obs} the reaction rate (moles MBY·kg catalyst$^{-1}s^{-1}$), D_e the diffusion coefficient (m^2s^{-1}), C_{MBY} the concentration of MBY at the catalyst surface ($mol{\cdot}m^{-3}$) and n the reaction order.[1,2]

The Weisz-Prater criterion was estimated for the core-shell catalyst, in which the reaction rate was the highest (2.75 moles MBY·$kg^{-1}{\cdot}s^{-1}$). The density of the catalyst was assumed to be the density of the Aerosil 50 support (100000 $kg{\cdot}m^{-3}$). The largest catalyst particles had a diameter of ~5 mm, as they were not pressed or sieved, and therefore we used this number for the calculation. We assumed the C_{MBY} at the catalyst surface to be equal to the concentration of MBY in the reactor (C_{MBY} =300 $mol{\cdot}m^{-3}$), as no external mass transfer limitations were present (Table S1). Since no experimental data of the diffusion coefficient of MBY in toluene was found, we used the diffusivity of n-pentanol in toluene (1.349 $\cdot 10^9$ m^2s^{-1})[3], which is a comparable compound. The result is <<0.08, therefore, we conclude that no internal mass-transfer limitations were present in the catalysts.

References

1 K. van der Borght, K. Alexopoulos, K. Toch, J. W. Thybaut, G. B. Marin and V. V. Galvita, *Catalysts*, 2019, **9**, 1–21.

2 P. . Weisz and C. . Prater, in *Advances in Catalysis; Frankenburg*, V.I.K.W.G., Rideal, E.K., Eds.; Academic Press: New York, NY, USA, 1, 1954, pp. 143–196.

3 G. Ramprasad, A. K. Mukherjee and T. R. Das, 1991, DOI: 10.1021/je00001a036.
